# Preschool growth and nutrition service – addressing common nutritional problems: a community based, primary care led intervention

**DOI:** 10.1080/17571472.2017.1391460

**Published:** 2017-11-03

**Authors:** Samantha Ross, Charlotte Wright

**Affiliations:** a School of Medicine, University of Glasgow (Guarantor), Glasgow, UK; b Community Child Health, School of Medicine, University of Glasgow and Honorary Consultant Paediatrician PEACH Unit, Royal Hospital for Children, Glasgow, UK

**Keywords:** Childhood obesity, health visitor, weight faltering, community management, GP education, GPwSI

## Abstract

Childhood obesity has been prioritised by the World Health Organization in a recent report, which calls for a holistic multiagency approach to tackling and reducing future risks of obesity and its associated co-morbidities. This article examines a health service approach to improving recognition and management of pre-school nutritional problems as part of training health care professionals. It explores the practicalities of setting up a local pathway for managing cases in the community with appropriate specialist support. This model, developed for the management of weight faltering, has now been adapted to tackle childhood obesity.

## Why this matters to me

As a GP and health professional with an interest in preschool nutrition, I believe that clinicians have a role in shaping services and contributing to multidisciplinary management. I believe we also have a role in adding to the evidence base in this relatively neglected area.

## Key messages

Nutritional problems in infants and young children are common and can be managed within primary care with access to specialist support services.

Adequate training of health professionals and GP education is key.

Service design should be embedded in local health services to ensure continuity of care and knowledge.

## Introduction and background

Improving Maternal and Infant Nutrition: A Framework for Action [1] (2011) states that ‘All those working with families with young children should have the knowledge and skills to give practical information and support on infant milk feeding, complementary feeding and establishing good eating patterns and/or signpost appropriately.’

Weight faltering is defined as a sustained drop in two weight centile spaces across two weighing intervals. Weight loss is common in infants and most cases are due to undernutrition. True weight faltering after 4 months is rare, seen in 0.5% infants [2]. However, temporary weight loss may occur commonly, for example due to illness, and recovers post illness. It is important to note that parents seek advice due to concerns about weight gain which can generate significant workload.

A randomised controlled trial carried out in Newcastle during the 1990s found that health visitor management (with access to a specialised community-based team) of weight faltering in children under 2 years led to better recognition and management, closer follow-up and significantly better long-term weight and height gain than conventional hospital-based management [3]. The term weight faltering has replaced ‘failure to thrive’ as the latter denotes both organic disease, and/or neglect, both of which were only seen in small numbers in this study. Only 5% of the cases seen were found to have organic disease and in the majority of cases weight faltering was due to undernutrition [4].

These results suggest that there could be a significant cohort of infants and young children being referred to secondary care for advice or even admission, where no underlying medical condition would be found. There was therefore the potential to manage nutritional issues effectively and provide reassurance within primary care while avoiding unnecessary investigations and reducing the burden on waiting times.

While weight faltering has long been the concern of heath visitors, overnutrition until recently has not been regarded as an issue of relevance. However childhood obesity is now increasingly acknowledged to be a significant public health challenge of the twentieth-first century. Over the last 50 years, childhood obesity prevalence has risen from around 5% in the 1960s to current levels: in Scotland, by 2014–2015, 9.8% of P1 children were noted to be at risk of obesity, and 21.8% at combined risk of overweight and obesity. 27–30-month data showed 17.5% were overweight, 7.6% obese, and 4.2% severely obese [5].

An increase in childhood obesity prevalence in England was recently noted [6].

Current thinking is that early identification and intervention in childhood obesity to effect weight-related behavioural changes (in terms of diet and physical activity), have the best chance of long-term reduction of prevalence and risk of associated co-morbidity such as diabetes, ischaemic heart disease, various cancers and non-alcoholic fatty liver disease.

Pre-school interventions available in the UK include HENRY (Healthy Exercise and Nutrition in the Really Young) [7], and MEND (Mind Exercise Nutrition Do it) and are based on parenting and behavioural change type interventions [8]. While these services have a role in treatment, the programmes may have limitations in responsiveness to local populations where there are specific issues such as high vulnerability and lack of engagement due in part to lack of parental perception [9]. Another disadvantage is the fragmented nature of these services, as however well planned, third sector involvement cannot be easily joined up and embedded into existing services, and costs prohibit universal training of staff.

## Service design

In 2002, a pilot community based service based on the Newcastle model commenced in Glasgow. Initially, funding from NHS Education Scotland enabled recruitment and training of two GPs with a special interest (GPwSI) in paediatric nutrition. Training was organised for liaison health visitors who supported colleagues in case recognition and early management of weight faltering. The model provided GP training and attachment to community paediatric clinics, with weekly case discussion and advice for health visitors.

Results showed good uptake in terms of education of health care professionals and recruitment of liaison health visitors, but drawbacks included low volume caseload per specialist health visitor and staff turnover, leading to loss of expertise.

A new proposal in 2013 advocated a smaller centralised team covering the entire health board area, allowing consolidation of expertise. Funding was secured via the Maternal and Infant Nutrition Framework. The new team comprises two growth and nutrition advisers with a background in health visiting and infant feeding and experience in delivering education on the UNICEF baby friendly initiative standards, a paediatric dietitian and a GPwSI in paediatric growth and nutrition.

The team’s remit is to train and educate health visitors and GPs in evidence-based best practice in infant and toddler feeding, case identification and management of common nutritional problems, and to provide one-to-one support for health visitor case management in the community. A virtual clinic allows weekly case discussion of cases either referred in by health visitors or GPs in primary care, or appropriately diverted from GP referrals to secondary care. Follow-up, if required, is at a multidisciplinary clinic allowing further assessment of cases not responding to initial advice or where there is a suspected disorder. A consultant paediatrician provides oversight to enable clinical governance standards to be met. This model has been used in other settings with success in resource management [10].

**Figure 1.**
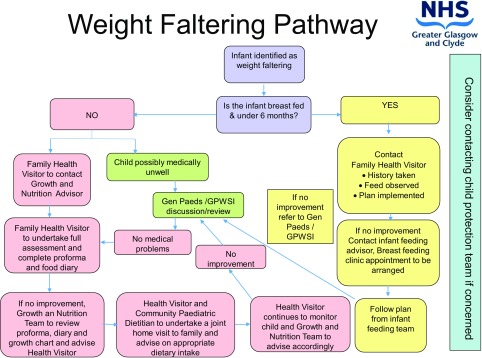
Weight Faltering Pathway in NHS Greater Glasgow and Clyde.

The service serves primary and secondary care and links to other health teams such as infant feeding, child protection and other specialist children’s services, acute services such as general paediatrics and tertiary services to allow prompt referral when required (Figure 1).

**Figure 2.**
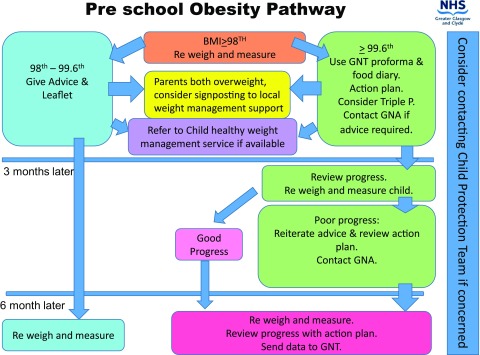
Preschool childhood obesity pathway in NHS Greater Glasgow and Clyde.

There is a cohort of children who are obese or severely obese and who might benefit from an integrated approach to management [5]. The model for weight faltering has been adapted to facilitate management of these cases (Figure 2). Health visitors identify obese pre-school children and can access the Growth and Nutrition Team for advice on managing behavioural change in the community. There has been an option for children who are severely obese (or who may have co-morbidity) to be referred on to the local specialist weight management service after initial work has been completed in the community. This is currently under review. Previously, overweight and obese toddlers were referred to and managed by community dietitians, but the rising prevalence of childhood obesity means there is a need for primary care staff to be skilled in assessment and early management. Staff need to be trained to recognise childhood obesity, raise the issue with families and encourage engagement. Empowering health visitors to manage cases helps to develop a family- partnership approach and overcome problems such as longer waiting times for a limited number of appointments with an expert but unfamiliar clinician.

The service commenced in 2014 and has expanded to include training and support for professionals in the management of suspected non IgE cow’s milk allergy as well as other issues related to paediatric nutrition such as faddy eating and managing constipation and reflux. Evaluation of the service and training has been positive. Link to results https://www.surveymonkey.com/results/SM-Y6FSQDKD8/ ( accessed 24 Oct 2017).

## Discussion

There is currently a recruitment crisis in specialist paediatric services, and more child health services may in the future be delivered by primary care practitioners. In particular, there is a need for service development focused on increasing recognition of childhood obesity and other nutritional issues. Appropriate referral management to allow management of selected cases crossing the interface of primary and secondary care allows for a safe, holistic and effective approach. The National Institute of Clinical Effectiveness (NICE) have recently published guidance which reflects our approach and has recommended service models which could be adapted to address identification and appropriate management of childhood obesity [11].

The role of a GPwSI in child health, or GP with extended roles (GPER) in aspects of child health could be developed and embedded within services to facilitate this approach, and is advocated by Modi and Simon [11, 12]. Infant and pre-school growth and nutrition is an area where community based management is feasible. A desirable outcome would be the development of UK-wide expertise in infant feeding generally and formalising the roles of GPs with a specialised or extended role in infant nutrition. Working with other primary-care-based health care professionals to ensure quality standards would facilitate this process. Increasing GP awareness and clinical expertise in the management of common nutritional problems could help to reduce inappropriate referrals to secondary care at a time of limited resources within the NHS. Informal GP networks have developed to meet the educational gap in this area [12, 13]. Challenges include ensuring stakeholder buy in, maintaining an effective and sustainable service and at a professional level ensuring appropriate accreditation and support for managing complex cases.

With scarce resources and competing priorities in the wider health service, as well as in primary care, it is important that health promotion and preventative strategies are not neglected. Medical education in infant and childhood nutrition requires greater emphasis as childhood obesity is becoming an increasingly important issue. The recently published Childhood Obesity Plan [13,14] has fallen short of expectations in addressing the obesogenic environment. NICE, the World Health Organization and others have placed prevention of childhood obesity as a health priority and have recommended action at national and local levels [15–18].

While GPs, health visitors and other primary care staff may be best placed to encourage behavioural change, lack of time, staff and expertise mean creative solutions need to be considered. Practitioners should provide messages on encouraging breastfeeding [19], responsive feeding, appropriate weaning and using portion sizes which help development of satiety and may help prevent obesity. Signposting families to available resources may be insufficient as vulnerable families with complex issues may not engage without support. Supporting staff development in the management of simple nutritional problems and allowing rapid access to appropriate care when required addresses WHO priorities within an existing framework, while building expertise and links with specialist services.

Limitations in the current model previously included the lack of a specific behavioural programme that can be undertaken by health practitioners in the pre-school obesity pathway. The team has now developed a targeted community intervention for hard-to-reach families where there is an affected obese child and where general health promotion through the obesity pathway has failed. This will be delivered by nursery nurses supported by specialist coaches allowing parenting and behavioural change approaches on healthy lifestyle to be explored. This could be embedded, following evaluation, into existing services.

We advocate a robust approach to commissioning training of professionals in nutrition across the UK and implementation by organisations – Public Health England have recently published a user guide to facilitate development of knowledge frameworks [20]. Improvements in education and service development ideally should involve GP champions for infant nutrition. Medical colleges (RCGP, RCPCH and FPH) would benefit from working in partnership to ensure appropriate standards for training and accreditation. Medical undergraduate education needs to include training on nutrition. Individual GPs can now access information locally and signpost appropriately with the launch of the GPIFN website [21]. A joined-up approach would lay the foundation for a preventative strategy to the obesity epidemic.

## Disclosure statement

No potential conflict of interest was reported by the authors.

## Governance information

NHS Greater Glasgow and Clyde.
